# Supplementary material for: Affect-focused psychodynamic psychotherapy for depression and anxiety through the Internet: a randomized controlled trial
Source: PeerJ. 2013 Jul 9;1:e102. doi: 10.7717/peerj.102 (PMC3709106; doi:10.7717/peerj.102)
Supplement: Supplemental Information 2 [file peerj-01-102-s002.pdf]

Research plan

Background

According to the National Council on Technology Assessment (2004) the economic costs of depression are high. A calculation performed in 1997 showed that the direct and indirect costs of depression went up to 12 billion in one year. Depression is very common compared to other medical diagnoses - an estimation is that 15-17% are affected at some time during their lifetime (Ebmeier, 2006). A large number of people with depression are not detected and those detected, are often given the wrong treatment (Kessler, 2003).

Both pharmacological and psychotherapeutic treatment has been shown to have documented efficacy in treating depression (SBU, 2004). There is some evidence that psychological treatment may result in fewer relapses in depression than medical treatment after 18 months of treatment (SBU, 2004). Internet-based CBT treatment of depression has been shown efficacy in previous studies (Andersson et al., 2005; Andersson & Cuijpers, 2009; Vernmark et al., 2010).

Our research group has previously conducted a first study of psychodynamic therapy delivered via the Internet, where the treatment has been shown to work for generalized anxiety disorder (GAD; Paxling et al., 2011). The study used a material that was based on a psychodynamic self-help book (Silverberg, 2005). Furthermore, a second study was conducted regarding depression (Johansson, et al., 2012). In both studies, the treatments had good effects, compared to control groups. In the present study, the material will be adapted to be suitable both for treating depression and anxiety. Further adaptation of the material will be made, based on an existing psychodynamic self-help book (Frederick, 2009).

Psychodynamic therapy has been shown to be effective in the treatment of depression (Driessen et al., 2010; Cuijpers et al., 2008). Other randomized trials have evaluated psychodynamic treatments for panic disorder (Milrod et al., 2007), generalized anxiety disorder (Leichsenring et al., 2009) and social phobia (Leichsenring, 2011). Based on this, it is reasonable to believe that the treatment that will be tested in the present format will be effective in the treatment of depression and anxiety disorders. Furthermore, previous studies on Internet-based psychotherapy, indicate that the treatment format also suits psychodynamic treatments. Based on these results, it is reasonable to assume that this approach can be adapted to be transmitted over the Internet.

The overall objective of this study is to investigate whether there are any differences in how the participants' symptoms of depression and anxiety are affected, as measured by self-report measures and clinical interview, when they either undergo psychodynamic Internet treatment or participate in the control group. The control group will receive weekly support sessions during the treatment period. After the treatment period, the participants from this group will be offered the same treatment as the treatment group has received.

The development of Internet-based cognitive behavioral therapy (CBT) has come a long way in Sweden and worldwide and is used today in the treatment of depression, anxiety disorders and in somatic conditions such as pain (Andersson, 2009). In Stockholm and Östergötland, there are ongoing studies involving patients referred to Internet-based CBT. An Internet-based treatment is manualised and based on text materials in modular form and may contain related exercises. The typical way to provide Internet-based treatment in Sweden is in the form of self-help texts supplemented by therapist assistance, ie the treatment is so-called guided self-help (Andersson et al., 2008). In a guided self-help therapy participants communicate with and receive feedback from a therapist, which distinguishes it from the computerized treatment program in that is has therapist support. It has been shown in meta-analyses of Internet-based depression treatment that guided self-help have a better effect than non-guided self-help (Andersson & Cuijpers, 2009).

Several studies have been conducted comparing CBT treatments that been delivered via the Internet. One aim has been to increase the availability of psychological therapy, to improve cost-effectiveness and reach out to people who do not seek traditional therapy (Andersson & Carlbring, 2003). Promising results support the continued use and development of the method. Carlbring and Andersson (2006) point out the importance of an accurate assessment at baseline, and a continuous monitoring of patient response to treatment.

Purpose and benefits of the study

The primary purpose of this study is to examine whether guided psychodynamic therapy over the internet works for participants with a diagnosis of major depressive disorder and anxiety disorders, and whether it provides clinically significant improvement compared to the control group.

Perhaps the main contribution the study could give is that it opens up for other treatment approaches. Psychodynamically inspired treatment method, presented in an understandable way, has not yet been tested for depression, and many clinicians identify themselves with a psychodynamic / psychoanalytic approach. Therefore, this study may become important.

Patient Selection and Procedure

The study will be advertised on the internet and in newspapers. After reading the information on our website, the participants notify their interest for the study. Subjects also sign a consent under the Personal Data Act (PUL) and sends it to the researchers. Outcome measures will be the PHQ-9 and the GAD-7. These measures will be filled out after application. People who meet inclusion criteria (having >10 on either the PHQ-9 or the GAD-7) will be contacted by telephone and asked to participate in a clinically structured interview. A psychiatrist will then analyze the screening results and the results from the structured interview. This is particularly important when medical conditions occur.

Individuals included in the study will be randomized to one of two conditions, one is a psychodynamic guided self-help treatment and the other is weekly support contacts. Past experience has indicated that the participants offered an active intervention while they are on

the waiting list has been estimated to maintain contact with the project. The control group will be offered the same treatment as the treatment group received, after the treatment period.

The treatment program consists of eight consecutive modules that participants work through with his/her therapist. Each module can be compared to a treatment session and will be administered at weekly intervals. Before starting treatment, all participants in both conditions need to fill out self-report measures forming baseline measurement. Each week will also require participants to fill out the PHQ-9 and the GAD-7.

The treatment is performed by a student from the last semester of training in the clinical psychologist programme, working under the guidance of an experienced psychotherapist with extensive clinical experience. Communication with clients is done via encrypted e-mail in a closed contact management system. All correspondence between the interviewer and clients will be filed in accordance with the patient act guidelines. Outcome measurements will be conducted after completion of the treatment, in addition to telephone interviews with all participants.

Overall structure for each weekly module:
1.Self-help text to be read by the participant.
2.Application of the material and application of the techniques in their own situation
3.Communication with therapist about the week's work.
4.Feedback from therapist.

Time schedule and Evaluation
1) January 2012: Modules will be revised and therapists are trained.
2) February 2012: Registration for the study. Applicants sign the consent under the Personal Data Act (PUL). Screening is completed.
3) February 2012: The first selection. Telephone interviews will be conducted. Discussion of interviews. Randomization. Messages will be sent to the people who have been excluded or are randomized to a treatment group or control group. Baseline measurement.
4) February-May 2012: Treatment.
5) May 2012: End Measurements. Follow-up interviews. Data collection.
6) May 2012: The control group will be offered treatment.

Project team
Gerhard Andersson, principal investigator of the study, Professor, lic psychologist and certified psychotherapist, Department of Behavioural Sciences and Learning, IBL, LiU
Robert Johansson, PhD student, lic. psychologist, IBL, LiU
Hugo Hesser, PhD student, lic. psychologist, IBL, LiU
Brjánn Ljótsson, PhD, lic. psychologist, Karolinska Institute, Stockholm
Andreas Rousseau, MD, Department of Psychiatry, Linköping

References

Andersson, G., Bergström, J., Holländare, F., Carlbring, P., Kaldo, V., & Ekselius, L. (2005). Internet-based self-help for depression: a randomised controlled trial. *British Journal of Psychiatry, 187*, 456-461.

Andersson, G. (2009). Using the Internet to provide cognitive behaviour therapy. *Behaviour Research and Therapy, 47*, 175-180.

Andersson, G., Bergström, J., Buhrman, M., Carlbring, P., Holländare, F., Kaldo, V., Nilsson-Ihrfelt, E., Paxling, B., Ström, L., & Waara, J. (2008). Development of a new approach to guided self-help via the Internet. The Swedish experience. *Journal of Technology and Human Services*, *26*, 161-181.

Andersson, G., & Carlbring, P. (2003). Editorial special issue. Internet and cognitive behavior therapy: New opportunities for treatment and assessment. *Cognitive Behavior Therapy*, *32*, 97-99.

Andersson, G., & Cuijpers, P. (2009). Internet-based and other computerized psychological treatments for adult depression: a meta-analysis. *Cognitive behaviour therapy*, *38*, 196-205.

Carlbring, P., & Andersson, G. (2006). Internet and psychological treatment. How well can they be combined? *Computers in Human Behavior*, *22*, 545-553.

Cuijpers, P., van Straten, A., van Oppen, P., & Andersson, G. (2008). Psychotherapy for depression in adults: a meta-analysis of comparative outcome studies. *Journal of Consulting and Clinical Psychology, 76*, 909-922.

Driessen, E., Cuijpers, P., de Maat, S., Abbass, A., de Jonghe, F., & Dekker, J. The efficacy of short-term psychodynamic psychotherapy for depression: a meta-analysis. *Clinical Psychology Review*, *30*, 25-36.

Ebmeier, K. P., Donaghey C., & Steele J. D. (2006). Recent developments and current controversies in depression. *Lancet, 367,* 153-167.

Frederick, R. (2009). Living like you mean it. San Francisco : Jossey-Bass.

Johansson, R., Ekbladh, S., Hebert, A., Lindström, M., Möller, S., Petitt, E., Poysti, S., Holmqvist-Larsson, M., Rousseau, A., Cuijpers, P., Carlbring, P., & Andersson, G. (2012). Psychodynamic guided self-help for adult depression through the Internet: a randomised controlled trial. *Unpublished manuscript.*

Kessler, R. C., Berglund P., Demler O., Jin, R., Koretz, D., Merikangas, K. R., Rush, A. J., Walters, E. E., & Wang, P. S. (2003). The epidemiology of major depressive disorder: results from the National Comorbidity Survey Replication (NCS-R). *Journal of the American Medical Association, 289,* 3095-3105.

Leichsenring, F. (2011). Psychotherapy for Social Phobia: First Results from the SOPHO-NET Study. *Abstract from the SPR 2011 Conference, Bern, Switzerland*

Milrod, B., Leon, A. C., Busch, F., Rudden, M., Schwalberg, M., Clarkin, J. , Aronson, A., Singer, M., Turchin, W., Klass, T., Graf, B. A., Teres, J. J., & Shear, M. K. (2007). A randomized controlled clinical trial of psychoanalytic psychotherapy for panic disorder. *American Journal of Psychiatry*, 164, 265-272.

Paxling, B., Almlöv, J., Dahlin, M., Carlbring, P., Breitholtz, E., Eriksson, T., & Andersson, G. (2011). Guided internet-delivered cognitive behavior therapy for generalized anxiety disorder: a randomized controlled trial. *Cognitive Behaviour Therapy*, 40, 159-173.

Statens beredning för medicinsk utredning. (2004). *Behandling av depressions-sjukdomar – En systematisk litteraturöversikt. Sammanfattning och slutsatser*. Stockholm: SBU.

Silverberg, F. (2005). Make the Leap. New York: Marlowe & Company.

Vernmark, K., Lenndin, J., Bjärehed, J., Carlsson, M., Karlsson, J., Öberg, J., Carlbring, P., Eriksson, T., & Andersson, G. (2010). Internet administered guided self-help versus individualized e-mail therapy: A randomized trial of two versions of CBT for major depression. *Behavior Research and Therapy*, *48*, 368-376.
